# Mechanisms of Copper Stress Response in Plants: Implications for the Medicinal Plant *Platycodon grandiflorus*

**DOI:** 10.3390/biology15120934

**Published:** 2026-06-15

**Authors:** Chi Liu, Shan Jiang, Junbai Ma, Meitong Pan, WenJing Sun, Denghua Wen, Ruoxi Zhang, Wei Ma, Xiubo Liu

**Affiliations:** 1 China National Teaching Instruments Import & Export Co., Ltd., Beijing 100032, China; liuchi@china-didac.com; 2 College of Pharmacy, Heilongjiang University of Chinese Medicine, Harbin 150040, China; 17390928032@163.com (S.J.); 15114516116@163.com (J.M.); meitong_pan@163.com (M.P.); 19104698212@163.com (W.S.); 18784213067@163.com (D.W.); 18363641002@163.com (R.Z.); 3 Jiamusi College, Heilongjiang University of Chinese Medicine, Jiamusi 154007, China

**Keywords:** *Platycodon grandiflorus*, copper stress, medicinal plant, antioxidant response, transcriptional regulation

## Abstract

Copper is an essential trace element for plant growth; however, in excessive amounts, it can cause severe toxicity by inducing bursts of reactive oxygen species and disrupting metabolic balance. As a root-based medicinal plant and food, *Platycodon grandiflorus* has its roots in direct contact with the soil. Its ability to accumulate copper is the most pronounced among various heavy metals; consequently, it is particularly susceptible to copper stress, which in turn affects its normal growth and medicinal quality. This paper focuses on the intrinsic stress potential and possible response pathways of *Platycodon grandiflorus* to copper stress. Drawing on existing research and relevant literature, it conducts an integrated analysis of its defence mechanisms across four levels: physical barriers, non-enzymatic antioxidants, conserved physiological and biochemical pathways, and transcriptional regulation. Regarding physical barriers, the cell wall forms the first line of defence through pectin adsorption and lignin deposition; in terms of endogenous antioxidant defence, secondary metabolites such as polysaccharides and saponins can directly participate in the scavenging of reactive oxygen species; regarding conserved pathways, the glutathione–phytochelate system acts in concert with antioxidant enzymes such as SOD and CAT to participate in copper ion chelation and the alleviation of oxidative stress, with hormone signalling regulation also playing a crucial coordinating role in this process; regarding transcriptional regulation, transcription factors such as PgWRKY may mediate the perception of stress signals and the expression of downstream genes. These pathways act in a coordinated and sequential manner, collectively forming a multi-level defence network through which *Platycodon grandiflorus* responds to copper stress. At the same time, this paper highlights the functional limitations of this defence system, summarises the shortcomings in current research, and proposes directions for future studies, with a view to guiding the safe cultivation and quality assurance of *Platycodon grandiflorus* in copper-polluted areas, as well as for the breeding of heavy-metal-tolerant medicinal plants.

## Simple Summary

Modern farming and industrial activities have left many farm soils contaminated with excess copper. This element is necessary for plant growth, yet too much of it will damage crops and ruin the quality of herbal plants. *Platycodon grandiflorus* is a popular plant used for food and traditional medicine. Since its roots grow underground, it easily absorbs excess copper from polluted soil. When copper-contaminated, the plant grows poorly and loses its medicinal value. We looked into how this plant resists copper-induced damage. Its root structure acts as a natural barrier to block copper. Some natural substances inside the plant can ease damage caused by copper. Multiple internal biological processes and genetic factors also work together to help the plant survive. We also pointed out the weaknesses of current studies and proposed new research directions. Our findings can guide farmers in growing safe, high-quality *Platycodon grandiflorus* on polluted land and help develop new herbal plant varieties that resist soil pollution.

## 1. Introduction

With the ongoing advance of industrialisation and the widespread use of agricultural chemicals, heavy metal contamination in soil has become increasingly severe, emerging as a key factor hindering the sustainable development of agriculture and threatening the ecological environment. Among the many heavy metal pollutants, copper has attracted widespread attention due to its dual biological effects [1,2,3]. On the one hand, copper is an essential trace element for plant growth and development; acting as a cofactor for numerous key enzymes, it plays a role in a range of core physiological processes, including photosynthesis, respiratory metabolism, carbon and nitrogen balance, and hormone signal transduction. On the other hand, when soil copper concentrations exceed the plant’s tolerance threshold, excess copper ions can have a marked toxic effect on plants [4,5]. High concentrations of copper not only interfere with the plant’s uptake and transport of other mineral elements but also catalyse the production of reactive oxygen species via the Fenton reaction, thereby causing severe oxidative stress in plants. This oxidative stress causes DNA damage, degradation of photosynthetic pigments and impaired cell wall synthesis in plants. It also disrupts the structure and function of key organelles, such as chloroplasts and mitochondria, ultimately inhibiting seed germination, hindering plant growth, and reducing biomass, whilst simultaneously diminishing the plant’s photosynthetic efficiency [6,7]. In addition, excessive copper can disrupt the homeostasis of the plant’s own antioxidant defence system, thereby further exacerbating cellular damage [8].

*Platycodon grandiflorus* is a traditional Chinese medicinal herb and a food; it is frequently used to treat respiratory conditions such as bronchitis and pneumonia. Its principal active constituents include *Platycodon grandiflorus* polysaccharides and saponins, which possess significant antioxidant, immunomodulatory, and anti-inflammatory properties [9,10]. In the main production areas of *Platycodon grandiflorus*, the long-term application of copper-based fungicides and high-copper-content manure has led to a continuous rise in soil copper levels. As the root is the medicinal part of *Platycodon grandiflorus*, it has a strong capacity to accumulate copper. Copper stress may not only inhibit the normal growth of *Platycodon grandiflorus* but also reduce its yield and visual quality. Still, it may also interfere with its secondary metabolic pathways by inducing oxidative stress. This can alter the patterns of synthesis and accumulation of key bioactive components, such as saponins and polysaccharides, thereby affecting their medicinal value [11,12,13]. Consequently, this study aims to elucidate the mechanisms underlying *Platycodon grandiflorus*’s stress response to copper, with a view to guiding the adoption of appropriate measures—such as breeding varieties with low copper accumulation or implementing soil improvement—to reduce copper uptake and enhance the safety of existing cultivation systems. Research has shown that when plants are subjected to copper stress, they typically activate their antioxidant defence systems to scavenge excess reactive oxygen species and maintain cellular redox balance [14,15,16]. However, when copper toxicity is severe, the protective role of the antioxidant system is limited; if the stress intensity exceeds the system’s capacity to neutralise it, the plant will still suffer oxidative damage [17]. *Platycodon grandiflorum* possesses strong antioxidant properties, and its polysaccharide components have been shown to alleviate oxidative stress and cellular damage caused by environmental pollutants. It is therefore hypothesised that when subjected to copper stress, *Platycodon grandiflorum* may also maintain cellular homeostasis by enhancing its endogenous antioxidant system [18,19,20]. This ensures the normal synthesis and accumulation of bioactive compounds. Furthermore, genomic studies have identified 42 WRKY transcription factors in *Platycodon grandiflorus*; these transcription factors belong to seven subfamilies, and their members exhibit distinct expression patterns under abiotic stress [21]. Given the key regulatory role played by the WRKY family in plants’ response to heavy metal stress, it is hypothesised that PgWRKY may also perform a similar function in *Platycodon grandiflorus*’s response to copper stress [22].

In recent years, research into the mechanisms underlying the plant response to copper stress has continued to advance, revealing the crucial roles of the MAPK signalling cascade, the copper transporter family, and copper-binding proteins in maintaining copper homeostasis and enhancing plant stress tolerance [23,24]. Preliminary studies on *Platycodon grandiflorus* have also revealed that the caffeoyl-CoA-O-methyltransferase gene family may be involved in the plant’s response to copper stress, and that this process is linked to lignin synthesis; this suggests that the reinforcement of the secondary cell wall may be one of the plant’s defence strategies against copper stress [25]. However, systematic research on *Platycodon grandiflorus*’s response to copper stress remains limited; in particular, there is a lack of in-depth analysis regarding the specific response patterns of its antioxidant system, the characteristics of metabolic reprogramming, and the upstream regulatory networks. Consequently, this review aims to systematically elucidate the physiological mechanisms by which copper stress induces toxicity in plants and to summarise the general response strategies of plant antioxidant defence systems. Furthermore, by focusing on *Platycodon grandiflorus*—a distinctive medicinal plant—this review integrates existing research evidence to explore its intrinsic stress response to copper stress and the associated molecular regulatory pathways. Due to the lack of direct experimental evidence in *Platycodon grandiflorus* under copper stress, this review integrates available data from this species with well-established mechanisms from model plants to propose testable hypotheses. By elucidating the response mechanisms of *Platycodon grandiflorus* to copper stress, we can not only ensure its safe cultivation and consistent quality in areas affected by heavy metal pollution but also provide theoretical support for the breeding of heavy metal-tolerant medicinal plants and their application in ecological restoration.

## 2. The Research Basis and Unique Characteristics of *Platycodon grandiflorus* Under Heavy Metal Stress

**Figure 1:**
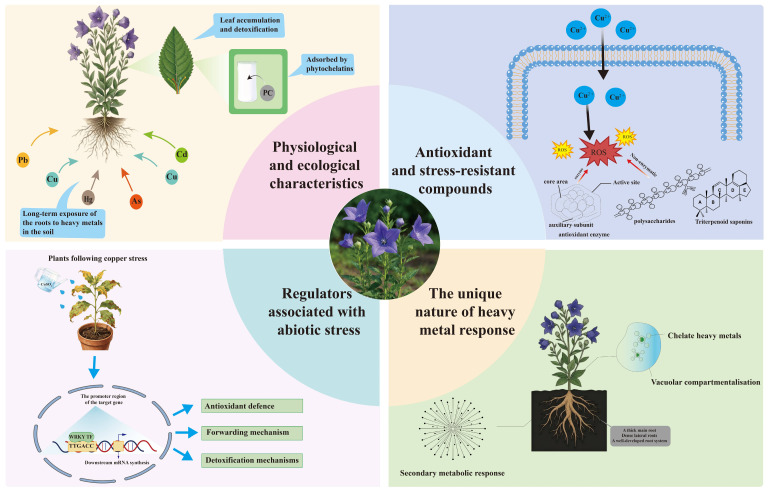
The Research Basis and Unique Characteristics of *Platycodon grandiflorus* under Heavy Metal Stress. This schematic is a simplified conceptual diagram that integrates the physiological, biochemical, and molecular mechanisms of *Platycodon grandiflorus* in response to heavy metal (mainly copper) stress. The whole figure is divided into four functional modules, and the scientific connotation of each part is described as follows: Physiological and ecological characteristics: As a root-harvested medicinal plant, *Platycodon grandiflorus* absorbs copper, lead, cadmium, and other heavy metals from the soil via its roots. Note: Phytochelatin (PC)-mediated copper chelation and vacuolar compartmentalization mainly occur in root cells, which act as the primary detoxification site. Only a small amount of residual copper is transported to leaves for secondary sequestration. Antioxidant and stress-resistant compounds: Enzymatic antioxidants, together with characteristic polysaccharides and saponins of *Platycodon grandiflorus*, eliminate copper-induced reactive oxygen species and alleviate oxidative stress. Regulators associated with abiotic stress: WRKY transcription factors recognize specific promoter sequences and activate downstream genes involved in defense, transport and detoxification pathways under heavy metal stress. The unique nature of heavy metal responses: Its robust root architecture and vigorous secondary metabolism endow *Platycodon grandiflorus* with distinct mechanisms of heavy metal tolerance.

As a perennial, root-bearing medicinal plant with dual medicinal and food uses, *Platycodon grandiflorus* relies on its underground roots as its primary medicinal part, which also exposes it to a high risk of stress in heavy metal-contaminated soils. Existing research indicates that *Platycodon grandiflorus* can accumulate and transport various heavy metals, including lead, cadmium, and copper, and is rich in bioactive compounds such as polysaccharides and saponins, suggesting its potential to alleviate oxidative stress induced by environmental pollutants. In recent years, transcription factors involved in responses to abiotic stresses, such as WRKY, have been systematically identified at the whole-genome level in *Platycodon grandiflorus*, laying a molecular foundation for elucidating its regulatory mechanisms under heavy metal stress. Compared with model plants, *Platycodon grandiflorus*’s unique secondary metabolic networks and root architecture may confer distinctive strategies for heavy-metal tolerance (Figure 1).

### 2.1. The Ecological and Physiological Characteristics of Platycodon grandiflorus as a Plant with Dual Medicinal and Culinary Uses

*Platycodon grandiflorus* is a traditional Chinese medicinal herb with both culinary and medicinal uses. The typical soil in the main growing regions of *Platycodon grandiflorus* is acidic to neutral (pH 5.5–7.0) with moderate organic matter content; sufficient base fertiliser must be applied during land preparation. In the cultivation of *Platycodon grandiflorus*, Bordeaux mixture is routinely used as a protective fungicide. However, long-term use of copper-based fungicides and copper-rich manure can lead to elevated total copper levels in the soil, sometimes even exceeding environmental safety thresholds [26]. Furthermore, soil properties (pH, CEC, organic matter) determine the bioavailability of Cu^2+^; lower pH values and higher levels of dissolved organic carbon enhance copper activity [27]. Because the medicinal part of the plant is its underground root system, which is in constant direct contact with the soil, it is at high risk of stress when cultivated in heavy-metal-contaminated areas. A survey of plants in the Daqing Mountain silver-lead mining area of Inner Mongolia conducted by Zhang Wei et al. found that *Platycodon grandiflorus* exhibits a certain capacity to translocate lead, cadmium, copper, zinc, nickel, and chromium, demonstrating its potential to respond to multiple heavy metals [28]. Research by Zhai Furong et al. on the soil-*Platycodon grandiflorus* system in the Xibo River basin, Chifeng, Inner Mongolia, further confirms that the medicinal parts of *Platycodon grandiflorus* tend to accumulate heavy metals, including copper [29]. A study measured the concentrations of five heavy metals—Pb, Cd, As, Hg and Cu—in different parts of *Platycodon grandiflorus* at various growth stages. The results indicated that *Platycodon grandiflorus* accumulates the highest levels of Cu (Nan, Meng et al.). These ecological characteristics mean that *Platycodon grandiflorus* is at high risk of exposure to heavy metal contamination in soil, whilst also making it a valuable medicinal plant for studying the mechanisms underlying heavy metal responses.

### 2.2. Known Antioxidant and Stress-Resistant Active Compounds in Platycodon grandiflorus

The main active components in *Platycodon grandiflorus* are primarily Platycodon polysaccharides and various saponin compounds. Research suggests that these compounds may alleviate oxidative stress induced by environmental pollutants [30]. For example, Platycodin D exhibits a relatively strong ability to scavenge peroxyl radicals. Furthermore, platycodin aglycone, desapiosidic platycodin E, Platycodin D, and Platycodin E showed scavenging abilities for peroxynitrite of 2.35, 1.27, 1.02, and 0.75 times that of glutathione, respectively. Furthermore, total *Platycodon grandiflorus* saponins significantly reduced malondialdehyde and reactive oxygen species (ROS) levels, whilst simultaneously increasing glutathione levels. Platycodin D, meanwhile, reduced the generation of ROS and malondialdehyde in damaged cells and enhanced the activity of superoxide dismutase and catalase. Turning to *Platycodon grandiflorus* polysaccharides, their maximum scavenging rates for hydroxyl radicals, ABTS cation radicals, and DPPH radicals reached 95.85%, 66.96% and 52.45%, respectively, demonstrating exceptionally strong antioxidant activity [31]. Furthermore, research has shown that *Platycodon grandiflorus* polysaccharides can effectively alleviate oxidative stress, inflammatory responses and cellular damage caused by environmental pollutants such as fumonisin B1 [18]. These results suggest that under copper stress conditions, in addition to the enzymatic antioxidant system, the non-enzymatic components naturally present in *Platycodon grandiflorum*—such as saponins and polysaccharides—may also exert a synergistic protective effect, helping maintain intracellular redox balance.

### 2.3. Identification of Regulatory Factors Associated with Abiotic Stress in the Platycodon grandiflorus Genome

In recent years, with advances in genomics technology, researchers have systematically identified the WRKY transcription factor family in *Platycodon grandiflorus*. A whole-genome analysis identified 42 *PgWRKY* genes in *Platycodon grandiflorus* and classified them into seven subfamilies using phylogenetic analysis. This study also integrated transcriptomic data to reveal that different members of the PgWRKY family exhibit distinct expression patterns under abiotic stresses such as cold, heat, and drought; for example, the expression level of *PgWRKY39* was significantly upregulated two days after drought treatment. Furthermore, the expression of some *PgWRKY* genes was altered upon methyl jasmonate induction, and they co-expressed with genes involved in the triterpenoid biosynthetic pathway, suggesting that four of these *PgWRKY* genes may regulate the synthesis of triterpenoid secondary metabolites. The study further identified key PgWRKY transcription factors that are responsive to temperature and drought stress, providing preliminary insights into the molecular mechanisms underlying the formation of *Platycodon grandiflorus* quality under abiotic stress [21]. The WRKY family comprises key regulatory factors in plants’ responses to abiotic stress, playing a significant role in the expression of antioxidant enzyme genes, the regulation of secondary metabolism, and signal transduction. Studies conducted in other species have also provided valuable insights into the function of *WRKYs* in *Platycodon grandiflorus*. For example, in *Triticum aestivum*, *TaWRKY74* is rapidly induced under copper stress, with its expression levels increasing more than 30-fold. It activates *TaGST1* expression by directly binding to its promoter region, thereby promoting glutathione accumulation. If *TaWRKY74* is transiently silenced, the expression of *TaGST1*, the activity of glutathione S-transferase, and glutathione levels all decrease markedly, whilst hydrogen peroxide, malondialdehyde and copper levels rise significantly. This indicates that it plays a key role in copper stress tolerance [32]. In *Oryza sativa*, *OsWRKY37* is strongly induced by copper deficiency; it upregulates copper uptake, as well as its transport and distribution between the roots and stems, by directly binding to and activating the promoters of the copper transporter genes *OsCOPT6* and *OsYSL16*, thereby maintaining copper homeostasis [22]. Although the mechanism by which WRKY responds to copper stress in *Platycodon grandiflorus* has not yet been explicitly reported, the cross-species evidence presented above strongly suggests that the PgWRKY family in *Platycodon grandiflorus* may also be involved in the regulatory network governing heavy metal (including copper) stress. Taken together, the WRKY transcription factor in *Platycodon grandiflorus* not only plays a regulatory role in traditional stress factors such as drought, cold, and heat, but may also influence its medicinal properties and environmental adaptability by regulating secondary metabolism and potential heavy-metal response pathways.

### 2.4. The Comparative Advantages of Platycodon grandiflorus over Other Plants in Heavy Metal Response

Compared with ordinary crops or model plants, *Platycodon grandiflorus*, as a perennial, root-based medicinal plant, may have developed a unique mechanism to tolerate heavy metals due to the structure of its root system and its metabolic characteristics. Research by Pang Ji and colleagues examined the effects of cadmium and mercury stress on the germination of *Platycodon grandiflorus* seeds and seedling growth. The results revealed that low concentrations of cadmium and mercury had little effect on seed germination potential and germination rate; however, as concentrations increased, the germination index and vitality index declined significantly. Furthermore, seedling root length, plant height, and fresh weight all decreased with increasing heavy metal concentrations [33]. In terms of physiology and biochemistry, the superoxide dismutase activity of *Platycodon grandiflorus* seedlings under cadmium and mercury treatment showed a trend of initially increasing and then decreasing as heavy metal concentrations rose, whilst peroxidase activity exhibited relatively complex fluctuations; Malondialdehyde levels initially decreased and then increased under cadmium stress, whereas they continued to rise under mercury stress; this indicates that the extent of heavy metal-induced oxidative damage is closely related to the intensity of the stress [33]. The polysaccharides and saponins abundant in *Platycodon grandiflorus* have been shown in in vitro studies to possess free-radical-scavenging activity. These compounds are likely to synergistically contribute to maintaining cellular redox homeostasis, building upon the enzymatic antioxidant system. Furthermore, *Platycodon grandiflorum* exhibits active secondary metabolic pathways, and there may be crossregulation between the synthesis of its bioactive compounds and stress responses. This provides some assurance that the plant can maintain its medicinal properties even in heavy-metal-contaminated environments.

### 2.5. Limitations of Existing Research and a Unique Approach to the Study of Platycodon grandiflorus

Although the mechanisms underlying plant responses to heavy metal stress have been extensively reported, research on *Platycodon grandiflorus*, a specific medicinal species, remains scattered. In particular, there is a lack of integrated analyses of its systemic physiological, biochemical and molecular responses to copper stress. Existing literature has largely focused on the effects of heavy metals such as cadmium, lead and aluminium on *Platycodon grandiflorus*, whilst research on copper—a unique heavy metal that is both essential and toxic—remains virtually non-existent. Research by Duan Yunjing et al. indicates that the ratio of nitrogen forms significantly influences the accumulation of trace elements such as copper, iron, manganese and zinc in *Platycodon grandiflorus* roots, with the highest copper accumulation observed under amide nitrogen treatment, suggesting that it is feasible to regulate the plant’s uptake of copper through cultivation measures [34]. A recent genome-wide analysis of the CCoAOMT gene family in *Platycodon grandiflorus* has provided new insights into the mechanisms underlying its response to copper stress. [25]. Research by Wang Ying and Zhang Zhuanglong has independently demonstrated that *Platycodon grandiflorus* polysaccharides can alleviate hexavalent chromium-induced apoptosis and oxidative damage, providing preliminary evidence for the protective role of endogenous antioxidants in Platycodon against heavy-metal stress [35,36]. Therefore, by drawing on the existing knowledge of *Platycodon grandiflorus*’s chemical constituents, genomic resources and ecological characteristics, and by conducting an in-depth analysis of its specific response mechanisms to copper stress, we can not only fill a knowledge gap in this field but also provide a scientific basis for the safe utilisation of medicinal plants in contaminated soils. It should be noted that the plant response to copper stress begins with the uptake of Cu^2+^ by the root system, primarily mediated by the high-affinity COPT/Ctr family and low-affinity non-specific cation channels. The strong ability of *Platycodon grandiflorus* roots to accumulate copper may be related to the high cation exchange capacity of their root cell walls and the high expression of copper transporters. However, copper uptake is not entirely selective; Cu^2+^ competes with Fe^2+^, Zn^2+^ and other ions for the same transmembrane transport systems, which is the primary cause of secondary nutrient imbalances induced by copper stress. To date, no reports have been published on the key transport genes involved in copper uptake in *Platycodon grandiflorus* or on their ion selectivity; this should therefore be an important area of research for elucidating the mechanisms underlying its heavy-metal accumulation.

## 3. General Physiological Toxic Effects of Copper Stress in Plants

Copper is an essential micronutrient for plants, playing a role in key physiological processes such as electron transport in photosynthesis, cell wall metabolism and signal transduction. However, excessive copper can cause significant toxicity in plants: it can directly damage chloroplast structure and impair the function of photosystem II, thereby inhibiting photosynthesis; it catalyses the generation of hydroxyl radicals via the Fenton reaction, triggering oxidative stress and membrane lipid peroxidation; it competitively inhibits the uptake and transport of essential elements such as iron and zinc, disrupting mineral nutrient homeostasis; and it interferes with hormonal balance, inhibiting seed germination, root development and reproductive growth.

### 3.1. Inhibition of Photosynthesis and Electron Transport

**Figure 2:**
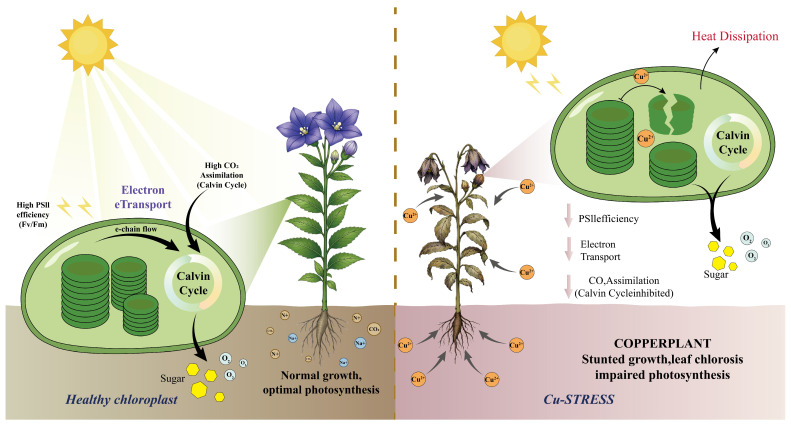
Inhibition of photosynthesis and electron transport. Schematic comparison of photosynthesis in healthy and copper-stressed *P. grandiflorus*. Under normal conditions (**left**), plants exhibit healthy chloroplast structure, with well-organized thylakoid stacks and intact photosynthetic apparatus. Light energy is efficiently captured by PSII, driving linear electron transport and supporting high CO_2_ assimilation via the Calvin cycle, ultimately promoting optimal photosynthesis and normal plant growth. Under copper stress (**right**), excess Cu^2+^ absorbed by roots is translocated to leaves and accumulated in chloroplasts. Cu^2+^ disrupts thylakoid membrane integrity and damages the photosynthetic machinery, leading to reduced PSII efficiency, impaired electron transport, and inhibition of the Calvin cycle. These functional disorders are accompanied by increased non-photochemical energy dissipation (heat dissipation) and reduced sugar production, ultimately resulting in stunted growth, leaf chlorosis, and severely impaired photosynthetic performance.

Copper stress can directly inhibit the function of the photosynthetic system, manifesting as decreased photosynthetic pigment content and impaired light-energy utilisation efficiency. Treatment with high concentrations of copper can significantly reduce chlorophyll a, chlorophyll b, and carotenoid levels in plant leaves, thereby compromising the stability of photosynthetic pigments [36]. In *Oryza sativa*, copper treatments at concentrations of 200 mg·kg^−1^ or higher resulted in a significant decrease in the photochemical efficiency of photosystem II, accompanied by increased non-photochemical quenching and unregulated energy dissipation, indicating impaired light energy capture and utilisation efficiency and energy wastage [37]. Studies on cowpea seedlings have also confirmed that, 48 h after copper stress treatment, the actual quantum efficiency of PSII, the rate of photosynthetic electron transport, and the photochemical quenching coefficient all decreased significantly [38]. At the structural level, excessive copper can disrupt the integrity of the chloroplast membrane system, leading to disordered thylakoid lamellar structure and loose grana stacking [36]. Regarding functional effects, copper stress inhibits the activity of the photosynthetic electron transport chain and disrupts carbon fixation. Feng Hanqing et al. found in their study of cowpea seedlings that, 48 h after copper stress treatment, the rate of photosynthetic electron transport decreased significantly, whilst the net photosynthetic rate fell further, indicating that the process of CO_2_ fixation via photosynthesis was markedly inhibited [38]. In their review, Shomali et al. systematically outlined the mechanisms by which metal stress regulates photosynthetic processes in plants, noting that the chloroplast electron transport chain is a primary target of metal stress, and that dysfunction in the carbon fixation process negatively regulates the activity of the electron transport chain; these two processes are interrelated and jointly influence photosynthetic efficiency [39]. Furthermore, copper can inhibit nitrate reductase activity, thereby affecting nitrogen assimilation and reducing the plant’s photosynthetic capacity (Figure 2).

### 3.2. Induction of Oxidative Stress and Membrane Lipid Peroxidation

**Figure 3:**
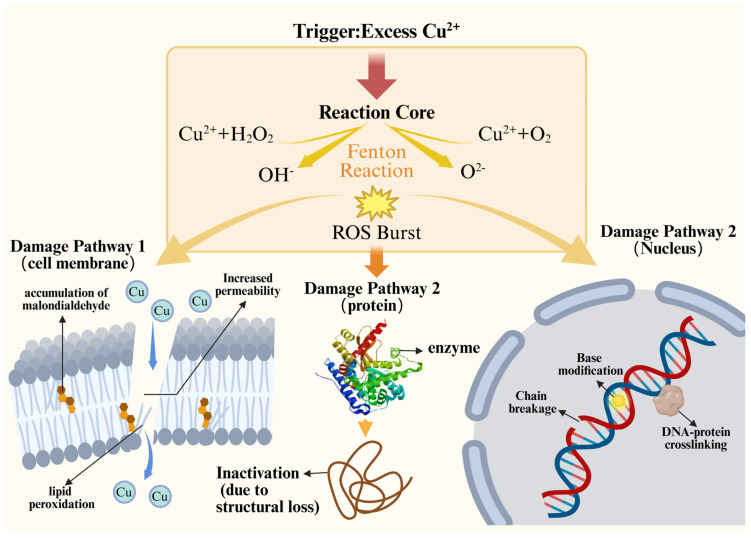
Induction of Oxidative Stress and Membrane Lipid Peroxidation. Excess Cu^2+^ acts as the trigger of cellular oxidative stress. As a transition metal, Cu^2+^ catalyzes the Fenton reaction with H_2_O_2_ to generate highly toxic hydroxyl radicals (OH^−^), and reacts with O_2_ to produce superoxide anions (O_2_^−^), leading to a burst of reactive oxygen species (ROS). Excessive ROS disrupts the cellular redox balance and causes multi-target damage to plant cells through three major pathways: Damage to the cell membrane (Damage Pathway 1): ROS attacks polyunsaturated fatty acids in the lipid bilayer, inducing lipid peroxidation. This results in increased membrane permeability and electrolyte leakage, accompanied by the accumulation of malondialdehyde (MDA), a marker of lipid peroxidation. Damage to proteins (Damage Pathway 2): ROS oxidize sulfhydryl groups on proteins, leading to protein denaturation and irreversible enzyme inactivation. Damage to nuclear DNA (Damage Pathway 2, nucleus): ROS entering the nucleus cause genetic toxicity, including DNA strand breaks, base modifications, and DNA-protein crosslinking. These cascading effects are the core mechanisms underlying copper-induced phytotoxicity.

Oxidative stress induced by excessive copper ions is one of the key mechanisms underlying its toxic effects. As a polyvalent metal, copper can catalyse the conversion of hydrogen peroxide into highly toxic hydroxyl radicals via the Fenton reaction, whilst also reacting with molecular oxygen to form superoxide anions [40]. The excessive production of these reactive oxygen species disrupts the cellular redox balance, triggering oxidative stress. The surge in reactive oxygen species causes widespread damage to cellular components, attacking unsaturated fatty acids in the biological membrane system and inducing membrane lipid peroxidation, which leads to increased membrane permeability and electrolyte leakage [36]. Malondialdehyde, a hallmark product of membrane lipid peroxidation, increases significantly under copper stress, reflecting the extent of damage to the cell membrane system. In *Trapa natans* leaves, the rate of reactive oxygen species production and malondialdehyde content gradually increase as the Cu^2+^ concentration rises. In plants such as *Oryza sativa* and *Brassica campestris*, increased malondialdehyde levels have been observed under copper stress, and these levels correlate positively with the severity of the stress (Htwe, Chotikarn et al. 2022) [37]. In *Oryza sativa* seedlings treated with 300 mg·L^−1^ of nano-copper oxide, malondialdehyde levels increased by 330.94%, hydrogen peroxide levels increased by 99.27%, and the superoxide anion generation rate rose by 159.92% [41]. Reactive oxygen species can also oxidise the thiol groups of proteins, leading to enzyme inactivation; once they enter the cell nucleus, they can induce genetic damage, such as DNA strand breaks, base modifications, and DNA-protein cross-links (Acila, Allioui et al. 2026) [41] (Figure 3).

### 3.3. Interfering with the Absorption and Transport of Mineral Nutrients

**Figure 4:**
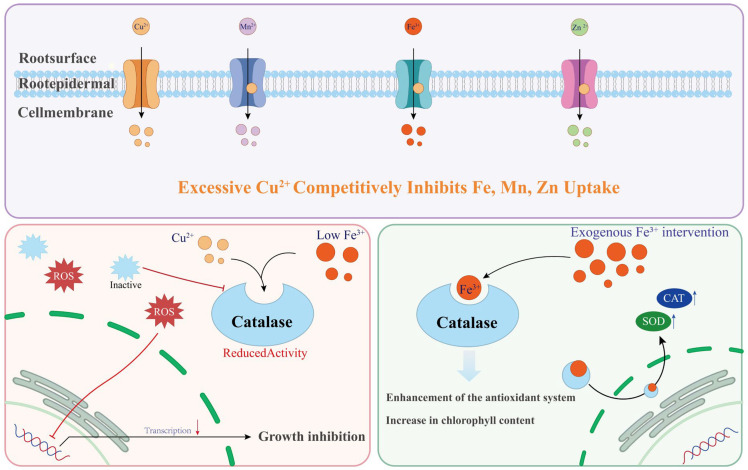
Interfering with the absorption and transport of mineral nutrients. The diagram illustrates the antagonistic interactions between excess Cu^2+^ and essential mineral elements (Fe, Mn, Zn), as well as their downstream physiological consequences and the mitigation effect of exogenous Fe supplementation. (**Top panel**) At the root surface, excess Cu^2+^ competes with Fe^3+^, Mn^2+^ and Zn^2+^ for non-specific binding sites on metal transporters, thereby competitively inhibiting the uptake and transport of these essential micronutrients and disturbing the plant’s mineral homeostasis. (**Bottom left panel**) Under Cu stress, reduced Fe availability leads to functional impairment of Fe-dependent enzymes such as catalase (CAT), resulting in decreased enzyme activity, increased reactive oxygen species (ROS) accumulation, inhibited transcription of growth-related genes, and ultimately plant growth inhibition. (**Bottom right panel**) Exogenous Fe^3+^ supplementation restores Fe availability, which enhances CAT activity, upregulates antioxidant enzymes (SOD, CAT), promotes chlorophyll synthesis, strengthens the antioxidant system, and alleviates copper-induced oxidative damage and growth inhibition.

There are actually quite complex interactions between excess copper ions and other mineral elements. High concentrations of copper inhibit the absorption and transport of essential trace elements such as iron, zinc, and manganese through a competitive mechanism, thereby triggering secondary nutrient deficiency symptoms [42]. For example, research has shown that in *Pinus radiata* seedlings, there is a clear antagonistic relationship between manganese and zinc uptake and copper uptake [42]. This interference is partly due to copper ions binding non-specifically to transport proteins for mineral elements, thereby occupying the binding sites intended for other metal ions and hindering their normal absorption. In Spinacia oleracea, the growth inhibition caused by copper stress is closely linked to the accumulation of copper in the leaves. It is accompanied by a disruption in overall mineral homeostasis [43]. This ion imbalance further disrupts the plant’s physiological metabolic processes, affecting the activity of key enzymes and cellular signalling. It is particularly important to note that under copper stress, iron levels within the plant decrease significantly, thereby impairing the function of iron-dependent enzymes. For example, catalase (CAT) is an iron-containing enzyme, and one possible reason for its reduced activity is impaired iron absorption. This view is also supported by a study on maize conducted by Kumar et al.: they found that under conditions of excessive copper exposure, catalase activity was significantly inhibited; however, when iron was supplemented externally, not only did the enzyme’s activity recover markedly, but the oxidative damage caused by copper stress was also alleviated [44]. This indicates that the decline in catalase activity under copper stress is closely linked to iron availability and highlights that the interaction between copper and iron plays a key role in regulating the plant’s antioxidant defence system (Figure 4).

### 3.4. Inhibiting Growth, Development and Reproductive Processes

**Figure 5:**
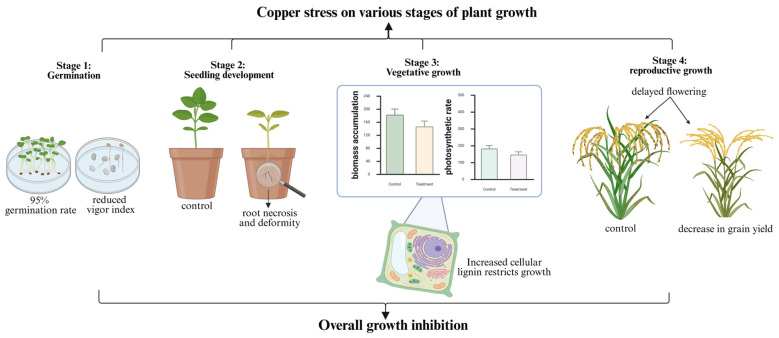
Inhibiting growth, development and reproductive processes. Copper stress exerts multi-stage inhibitory effects on plant growth from seed germination to reproductive development. This diagram systematically summarizes the key impacts at each stage: Germination stage: Seeds are highly sensitive to copper toxicity. High copper concentrations significantly reduce the germination rate and seed vigor index. Seedling development stage: Excess copper causes severe root damage, including root necrosis, deformity, and reduced root surface area and volume, which impairs water and nutrient uptake. Shoot growth is also inhibited, with reduced shoot length and biomass accumulation. Vegetative growth stage: Copper stress suppresses photosynthetic rate and biomass accumulation. Increased cellular lignification restricts cell expansion, further limiting plant growth. Reproductive growth stage: Copper exposure leads to delayed flowering, reduced leaf area, and a significant decline in grain yield. Collectively, these stage-specific responses result in overall inhibition of plant growth under copper stress.

The most obvious effect of copper stress on plants is inhibition of growth and development; indeed, a marked inhibitory effect is evident across multiple stages, from seed germination to reproductive growth. The seed germination stage is particularly sensitive to copper stress; treatment with high copper concentrations significantly reduces seed germination potential, germination rate, and the vitality index [41]. Once the seedlings have emerged, the symptoms of damage become more pronounced, primarily manifesting as stunted root and shoot growth and reduced fresh and dry weight. When *Oryza sativa* seedlings were treated with 300 mg·L^−1^ of nano-copper oxide, shoot length decreased by 31.89%, whilst root length decreased by as much as 98.08% [45]. As the organ directly exposed to copper stress, the root system is severely affected in its morphological development; the number of root tips, root surface area, and root volume are all significantly reduced, and root tips often exhibit browning, deformities, and even necrosis. This damage to the root system further limits the plant’s ability to absorb water and mineral nutrients, thereby exacerbating the overall growth-inhibiting effect [46]. The inhibitory effect of copper stress on plant height growth and biomass accumulation was equally pronounced. In a pot experiment, a copper treatment at 100 mg·kg^−1^ reduced plant height by 19.7% and decreased the photosynthetic rate by 17.3% [47]. This is partly due to restricted photosynthesis and partly to increased lignification of the cell walls, the latter of which limits normal cell expansion [48]. During the reproductive growth stage, when *Oryza sativa* is treated with copper at concentrations of 200–400 mg·kg^−1^, not only does leaf area decrease and flowering time be delayed, but grain yield also falls significantly, by between 26% and 71% [37] (Figure 5).

### 3.5. Hormone Response and Signal Transduction

Excessive copper stress has a marked toxic effect on plants, and one of the key mechanisms involved is interference with hormonal homeostasis and signal transduction networks. Copper stress affects growth-related hormones; for example, the accumulation of auxin (IAA) and cytokinin (CTK) is inhibited, and the associated signal transduction pathways are disrupted. This manifests directly as impaired root elongation and slowed growth of the aboveground parts [49]. Plants activate certain self-protective mechanisms in response; a typical example is a marked increase in abscisic acid (ABA) levels. ABA activates the expression of a series of antioxidant genes (such as GPX), helping cells scavenge excess reactive oxygen species generated by copper toxicity, thereby mitigating damage to the membrane system [50]. At the molecular level, several signal transduction events have also been implicated in the response to copper toxicity. In studies of plants such as *Solanum Lycopersicum*, copper treatment leads to the upregulation of a key enzyme (ABA 8′-hydroxylase) in the ABA metabolic pathway, which may help maintain ABA at appropriate levels; simultaneously, the BAK1 gene, which is associated with cell wall synthesis and stress tolerance, is downregulated, a change thought to be related to copper-induced programmed cell death [50]. Furthermore, ethylene signalling is somewhat unique, as the ethylene receptor protein itself requires copper ions as a cofactor; when copper levels are excessive, the receptor’s normal function is disrupted, which in turn prompts the plant to produce more stress-induced ethylene, creating a specific feedback loop [50]. Genes involved in the brassinosteroid (BR) signalling pathway (such as BR1 and BR2) also exhibit changes in expression, suggesting that multiple hormonal signalling pathways do not act in isolation but rather interact synergistically [49]. These signals from different hormones ultimately converge with the universal MAPK cascade within the cell, together forming a complex regulatory network that helps plants survive as long as possible under conditions of copper toxicity.

## 4. General Overview of Antioxidant Systems in Plant Copper Stress Responses

**Figure 6:**
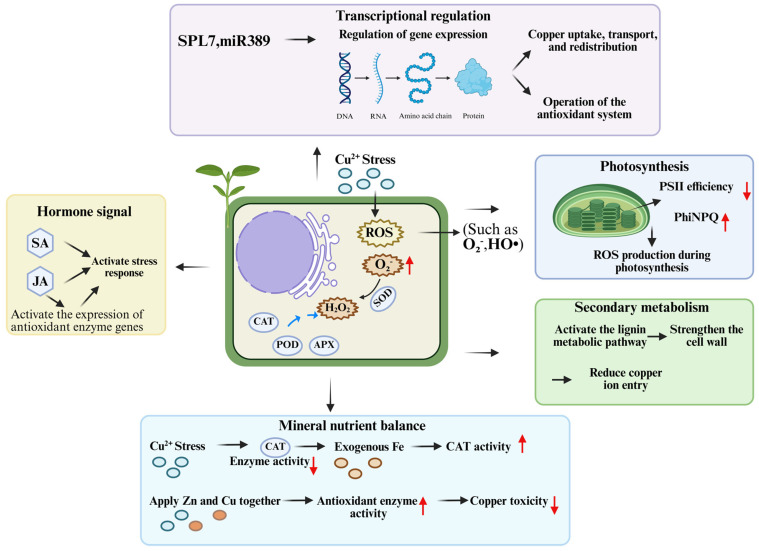
An Overview of the Current State of Research on the Role of Antioxidant Systems in Resisting Copper Stress in Plants. Copper stress induces excessive accumulation of reactive oxygen species (ROS) and oxidative damage in plant cells. To counteract this, plants deploy multi-layered antioxidant defense systems: enzymatic antioxidants (SOD, CAT, POD, APX) and hormone signaling (SA/JA) work together to scavenge ROS and activate stress responses. Transcriptional regulators, such as the miR389-SPL7 module, further control the expression of genes involved in copper transport and antioxidant function. Copper stress also impairs photosynthesis, while secondary metabolism strengthens cell walls to reduce copper entry. Balanced mineral nutrition (e.g., Fe/Zn supplementation) restores enzyme activity and alleviates copper toxicity. These interconnected mechanisms form a comprehensive network that enables plants to resist copper-induced oxidative stress.

Under copper stress, excessive accumulation of reactive oxygen species (ROS) within plants causes oxidative damage to cellular structures. To counteract this damage, plants have evolved a complex antioxidant defence network. This network primarily comprises two major categories of systems: enzymatic and non-enzymatic antioxidant systems. The former, centred on superoxide dismutase, catalase, peroxidase, and ascorbate peroxidase, works synergistically to scavenge ROS; the latter encompasses glutathione, ascorbic acid, plant chelating peptides, and secondary metabolites such as phenolics and flavonoids, which perform both free-radical scavenging and metal-chelation functions (Figure 6).

### 4.1. Activation and Functional Differentiation of the Antioxidant Enzyme System

To cope with copper-induced oxidative stress, plants typically activate their antioxidant enzyme systems, which primarily comprise superoxide dismutase (SOD), catalase (CAT), peroxidase (POD), and ascorbate peroxidase (APX). In this process, SOD acts as the first line of defence, rapidly converting superoxide anion radicals into hydrogen peroxide and oxygen; the resulting hydrogen peroxide is then eliminated by enzymes such as CAT, POD and APX. Studies have shown that in *Halophila beccarii*, copper stress significantly increases the activity of SOD and CAT, thereby helping to scavenge excess reactive oxygen species and mitigate cellular damage [51], A similar phenomenon has been observed in *Vitis vinifera*, *Oryza sativa* and cucurbits, where copper treatment similarly induces an increase in the activity of enzymes such as SOD and POD, thereby forming a defensive barrier [52,53,54]. However, some studies have found that changes in the activity of these enzymes under copper stress are closely related to the severity of the stress and the duration of treatment. Once the copper concentration exceeds a certain threshold, the activity of antioxidant enzymes may actually be inhibited. For example, under high-copper treatment, the activities of APX and guaiacol peroxidase (GPX) in Cucurbita pepo decreased, suggesting that the antioxidant system may be experiencing functional saturation or an imbalance [55]. Another study on *Helianthus annuus* root systems showed that, following treatment with 50 μM CuSO_4_, SOD activity decreased. In contrast, CAT and guaiacol peroxidase activities increased significantly, indicating that different enzyme classes respond differently to copper stress [56]. This pattern of change also suggests that the protective capacity of the antioxidant enzyme system is limited. When the intensity of stress exceeds their ability to neutralise it, the enzyme proteins themselves may also suffer oxidative damage, leading to a functional imbalance in the entire system.

### 4.2. The Synergistic Defensive Effects of Non-Enzymatic Antioxidants

In response to copper stress, plants rely not only on enzymatic antioxidant systems but also on a variety of non-enzymatic antioxidants, which work in concert to defend against oxidative damage. Among these, glutathione (GSH) is a typical small-molecule peptide antioxidant; it not only directly scavenges reactive oxygen species (ROS) but also provides the essential reducing power needed to regenerate ascorbic acid (AsA) within the ascorbic acid-glutathione (AsA-GSH) cycle. Studies have shown that, under copper stress, the levels of AsA (ascorbic acid) and GSH (reduced glutathione), as well as the total antioxidant capacity (T-AOC), in *Elsholtzia splendens* from mining areas are significantly higher than in those from non-mining areas, indicating that the accumulation levels of these small-molecule antioxidants are closely related to the plant’s tolerance to copper [57]. Furthermore, although plant chelating peptides cannot directly scavenge reactive oxygen species, their synthesis relies on glutathione as a substrate; by chelating free Cu^2+^, they can reduce copper-induced cytotoxicity, thereby providing indirect protection [58]. At the same time, some secondary metabolites serve as both antioxidants and metal chelators. For example, soy isoflavones play a key role in the defence of *Glycine max* against copper toxicity [59]; Under copper stress, the total phenolic and flavonoid contents in *Marrubium vulgare* also increased significantly [60], The accumulation of these non-enzymatic antioxidants, together with the activation of antioxidant enzymes such as superoxide dismutase (SOD) and catalase (CAT), forms part of its comprehensive defence mechanism. Further studies have revealed that the exogenous application of certain signalling molecules or metabolic modulators can also effectively enhance plants’ tolerance to copper stress. For example, following treatment with 5-aminoacetylpropionic acid (ALA), the activity of antioxidant enzymes such as SOD and peroxidase (POD) increases significantly, whilst the concentration of malondialdehyde (MDA), a product of membrane lipid peroxidation, decreases. This indicates that regulating the metabolism of non-enzymatic antioxidants is an effective approach to alleviating copper toxicity [61]. Furthermore, the chemical form of copper significantly affects its toxicity and the plant’s antioxidant system response. Compared with conventional copper salts (such as CuSO_4_), nano-copper (nano-Cu) accumulates to a lesser extent within plants—by day 7, the copper content in leaves treated with 100 mg L^−1^ of nano-Cu was 76% lower than that in leaves treated with CuSO_4_, and the resulting oxidative stress response was also relatively milder [62]. This suggests that different forms of copper exert varying levels of oxidative stress on plants, thereby influencing the degree of activation and the defensive efficacy of non-enzymatic and enzymatic antioxidant systems.

### 4.3. Integrated Regulation of the Antioxidant System and Other Physiological Processes

At the regulatory level, the activation of the plant antioxidant system is precisely coordinated by a complex signaling network. Recent studies have shown that the transcription factor SQUAMOSA promoter-binding protein-like 7 (SPL7), as well as microRNA families associated with copper response (such as miR398), play a central role in maintaining copper homeostasis. By finely regulating gene expression networks, they coordinate the plant’s adaptive response to changes in copper levels. These regulatory factors not only participate directly in copper uptake, transport, and redistribution but also indirectly influence the operational efficiency of the antioxidant system [63,64]. Antioxidant defence does not operate in isolation, but is highly integrated with physiological processes such as hormonal signalling, mineral nutrient balance, photosynthesis and secondary metabolism. For example, under copper stress, the levels of jasmonic acid (JA) and salicylic acid (SA) rise significantly; these compounds likely regulate the expression of antioxidant enzyme genes through signal transduction pathways, thereby mediating the plant’s adaptation to stress [65]. This hormone-mediated regulatory mechanism enables plants to effectively integrate external environmental signals with internal defence responses. In mineral nutrition, copper stress often interferes with the uptake and transport of other essential elements, such as iron and zinc. The interaction between iron and copper is particularly crucial in regulating antioxidant enzyme activity: catalase (CAT) is an iron-containing enzyme, and a decline in its activity is partly due to copper stress, which hinders iron uptake; however, exogenous iron supplementation can restore CAT activity and alleviate oxidative damage. Similarly, the co-application of zinc and copper can enhance antioxidant enzyme activity and mitigate copper toxicity, highlighting the importance of interactions among mineral elements in regulating oxidative stress [55]. In terms of photosynthetic physiology, copper stress significantly inhibits the efficiency of photosystem II (as evidenced by decreases in Fv/Fm and Phi2), whilst simultaneously enhancing non-photochemical quenching (PhiNPQ). This regulation reflects the plant’s strategy of dissipating excess light energy as heat, thereby reducing the production of reactive oxygen species (ROS) in the photosynthetic electron transport chain and forming a synergistic protective mechanism with the antioxidant system [48]. Furthermore, activation of lignin metabolic pathways may limit copper ion entry into the cytoplasm by strengthening the cell wall, thereby indirectly reducing the oxidative burden within the cell [48]. This multi-level defence strategy, ranging from the cell wall to the chloroplast, clearly demonstrates how plants integrate multiple physiological processes to form a complex regulatory network in response to copper stress.

### 4.4. The Enhancing Effects of Exogenous Regulatory Mechanisms on the Antioxidant System

Given that plants’ intrinsic antioxidant capacity has functional limitations under severe copper stress, researchers have begun exploring exogenous interventions to enhance copper tolerance, with significant progress already made. Regarding the regulation of hormonal substances, studies have found that exogenous addition of 500 μmol·L^−1^ melatonin can significantly enhance the antioxidant enzyme activity in Oryza sativa seedlings, with SOD, POD, and CAT increasing by 17.51%, 34.54%, and 137.33%, respectively, whilst MDA content decreased by 38.04%. whilst H_2_O_2_ levels and the rate of superoxide anion generation were also reduced [7]. Similarly, in *Triticum aestivum*, treatment with aliphatic amines effectively enhanced the activity of key enzymes in the ascorbic acid-glutathione cycle (such as APX, GR and DHAR), and increased the AsA/DHA and GSH/GSSG ratios. Consequently, the accumulation of MDA and electrolyte leakage was reduced, and cell membrane integrity was maintained [66]. About the regulation of mineral elements, the application of silicon (Si) and the rare earth element cerium (Ce) has also been shown to alleviate copper toxicity. For example, in *Dendrobium nobile*, the addition of cerium can significantly mitigate the adverse effects of copper stress by regulating the expression of genes associated with antioxidant defence mechanisms and heavy-metal chelation [67]. Silicon treatment, however, can significantly improve plant biomass and various physiological parameters under copper stress; its mechanism of action includes enhancing the activity of antioxidant enzymes and reducing copper transport to the plant’s aboveground parts. Furthermore, L-glutamic acid (L-Glu) has been shown to alleviate copper-induced oxidative damage by modulating the antioxidant system [68]. In addition to the intervention of these exogenous substances, transgenic strategies have also demonstrated considerable potential for application. Studies have shown that increasing endogenous ascorbic acid (AsA) content in plants can effectively enhance their tolerance to copper stress, highlighting the value of antioxidant metabolic engineering in improving plant stress resistance [69]. Overall, enhancing the function of the antioxidant system through the regulation of exogenous substances or metabolic engineering has become an important technical approach for mitigating copper toxicity.

### 4.5. The Competitive Effects of Divalent Metals on Antioxidant Enzyme Activity Under Copper Stress

Under copper stress in plants, various divalent metals competitively affect the activity of antioxidant enzymes. Iron (Fe) is an essential cofactor for catalase (CAT), and excess copper inhibits the uptake and transport of iron. In *Marrubium vulgare*, copper stress reduced Fe^2+^ uptake in a concentration-dependent manner [60]; in *Zea mays*, CAT activity was inhibited under copper stress, whereas exogenous iron supplementation (500 μM) restored enzyme activity and enhanced antioxidant defence capacity, whilst significantly alleviating copper-induced oxidative damage [44]; similarly, zinc (Zn) and manganese (Mn) affect different isoenzymes of superoxide dismutase (SOD), respectively, and copper interference with zinc and manganese distribution inhibits the activity of this enzyme. In cereal crops (wheat and barley), excess copper significantly downregulated the expression of the MnSOD gene [70]. Calcium (Ca) indirectly regulates POD and CAT via signalling pathways; in soybean seeds, 4.5 mmol/L exogenous calcium increased POD activity under copper stress whilst decreasing CAT activity, with the two forming a coordinated relationship. Magnesium (Mg) exerts a general protective effect on SOD, CAT and ascorbate peroxidase (APX), and magnesium deficiency significantly exacerbates copper toxicity. Under adequate magnesium supply, the activities of SOD, APX and CAT in wheat are restored, and the levels of H_2_O_2_ and MDA decrease significantly. Furthermore, the addition of cations such as Ca^2+^ and Mg^2+^, which compete for metal-ion binding sites, can effectively prevent excessive accumulation of copper within cells. These antagonistic interactions among metals collectively determine the defensive efficiency of the antioxidant system [71].

## 5. The Intrinsic Stress Potential and Possible Response Pathways of *Platycodon grandiflorus* to Copper Stress

Under copper stress, plants maintain cellular homeostasis through a multi-level defence system. As a root-based medicinal plant, *Platycodon grandiflorus* forms a first line of defence through its cell walls, where pectin binding and lignin deposition limit the influx of copper ions. Concurrently, endogenous compounds such as polysaccharides and saponins, which are abundant in *Platycodon grandiflorus*, possess both free-radical-scavenging and membrane-protective functions and can work in concert with the enzymatic antioxidant system to mitigate oxidative damage. At the intracellular detoxification level, the glutathione–phytochelate pathway and compartmentalisation further reduce the toxicity of free copper. These processes are precisely regulated by transcription factors such as WRKY, forming a complete network spanning from signal perception to functional execution. The various response mechanisms dynamically coordinate throughout the stress response, collectively establishing *Platycodon grandiflorus*’s defence system against copper stress.

### 5.1. The Barrier Function and Structural Modifications of the Cell Wall

As a root-based medicinal herb also used as food, *Platycodon grandiflorus* has cell walls that act as the first line of defence against copper ion entry. These cell walls are primarily composed of cellulose, hemicellulose, pectin and lignin. Pectin, in particular, is rich in free carboxyl groups, which can bind to positively charged copper ions through electrostatic interactions, thereby trapping the copper within the cell walls. This reduces the amount of copper entering the cell interior, thereby helping to mitigate its toxicity [72]. In addition, the cell wall can enhance its barrier function through dynamic modifications. Previous studies have identified a family of caffeoyl-CoA-O-methyltransferase (CCoAOMT) genes in *Platycodon grandiflorus*; members of this family play a key catalytic role in the synthesis of lignin monomers [25]. In addition, the cell wall can enhance its barrier function through dynamic modifications. Previous studies have identified a family of caffeoyl-CoA-O-methyltransferase (CCoAOMT) genes in *Platycodon grandiflorus*; members of this family play a key catalytic role in the synthesis of lignin monomers and are involved in responses to copper stress [72]. Based on this, it can be inferred that under copper stress, *Platycodon grandiflorus* may promote lignin accumulation in root cell walls by upregulating expression of relevant genes in this family, thereby forming a denser physical barrier. As the medicinal part of *Platycodon grandiflorus* is its root, and since the root is in constant contact with the soil, increasing the binding capacity of copper by raising the levels of pectin and hemicellulose, combined with promoting lignin deposition to limit copper mobilisation, are likely to be key adaptive strategies for coping with copper stress [73]. Future research could focus on the expression patterns of key lignin synthesis genes, such as *PgCCoAOMT*, under copper treatment, as well as their relationship to copper distribution within plants, thereby providing theoretical support for ensuring the safety of medicinal materials.

### 5.2. The Direct Involvement of Endogenous Antioxidants

*Platycodon grandifloras* differs somewhat from typical crops; one of its most notable characteristics is that its roots are rich in two types of secondary metabolites: polysaccharides and saponins. Under normal conditions, these compounds have been shown to scavenge free radicals, chelate metal ions, and stabilise cell membrane structures; consequently, they are likely to play a direct role in antioxidant defence mechanisms during copper-induced oxidative stress. Research has shown that Platycodon polysaccharides are highly effective at scavenging hydroxyl radicals, ABTS cation radicals and DPPH radicals. This ability to scavenge free radicals may be activated under copper stress conditions, acting as part of a non-enzymatic antioxidant system to help eliminate reactive oxygen species (ROS) generated by the Fenton reaction [74,75]. At the same time, the saponins found in *Platycodon grandiflorus*, particularly Platycodon saponin D, have also been shown to reduce levels of reactive oxygen species and malondialdehyde (MDA) significantly in damaged cells, whilst simultaneously upregulating the activity of superoxide dismutase (SOD) and catalase (CAT) [76]. This suggests that saponins not only possess direct antioxidant properties themselves, but may also enhance the activity of the enzymatic antioxidant system via certain signalling pathways. Although there are currently no direct studies conclusively confirming that the polysaccharides and saponins in *Platycodon grandiflorus* are involved in the plant’s stress response to copper stress, based on the antioxidant effects they have demonstrated in other toxicity models [77], It is reasonable to infer that, under copper-stress conditions, these two types of compounds may also play a potential protective role in alleviating the accumulation of reactive oxygen species and inhibiting membrane lipid peroxidation.

### 5.3. Conservative Physiological and Biochemical Stress Pathways

In addition to being rich in unique compounds such as polysaccharides and saponins, *Platycodon grandiflorus* likely also utilises copper stress response mechanisms common throughout the plant kingdom to combat copper toxicity. In terms of intracellular detoxification, glutathione (GSH) and its derivatives, phytochelates (PCs), play a crucial role in chelating excess copper ions and sequestering them into vacuoles [78,79,80]. The efficiency of this detoxification mechanism depends largely on the plant’s ability to regenerate and recycle glutathione. Previous studies have shown that total saponins from *Platycodon grandiflorus* can significantly increase glutathione levels. This suggests that under copper stress, *Platycodon grandiflorus* may maintain glutathione homeostasis through its unique constituents, thereby ensuring that the detoxification process mediated by plant cells proceeds normally [81]. At the same time, the synergistic upregulation of the antioxidant enzyme system (SOD, CAT, POD, APX) can effectively scavenge copper-induced superoxide anions and hydrogen peroxide, thereby preventing excessive accumulation of reactive oxygen species and the resulting cellular damage. Furthermore, certain hormonal signals, such as abscisic acid (ABA), jasmonic acid (JA) and salicylic acid (SA), also accumulate significantly under copper stress; they may enhance plant tolerance to copper stress by regulating stomatal closure, the expression of antioxidant enzymes, and the reprogramming of secondary metabolism [65]. The *PgDXR* gene, which is involved in the synthesis of terpenoid precursors in *Platycodon grandiflorus*, has been shown to exhibit a marked transcriptional response to treatment with ABA, SA and methyl jasmonate; this suggests that the secondary metabolic network in *Platycodon grandiflorus* is likely to be crossregulated with the hormone-mediated defence pathway against copper stress [82]. In addition to the mechanisms described above, it remains to be investigated whether the metallothionein genes identified in *Platycodon grandiflorus* also play a role in the chelation and compartmentalisation of copper ions; this is a direction worthy of further exploration in future research.

### 5.4. The Potential Role of Transcriptional Regulatory Networks

Transcription factors play a central role in coordinating plant stress responses, and the 42 WRKY transcription factors identified in the *Platycodon grandiflorus* genome provide a rich reservoir of regulatory elements for the plant’s response to copper stress [21]. Members of the WRKY family can specifically recognise W-box elements in the promoter regions of target genes, thereby regulating the expression of downstream functional genes, including antioxidant enzyme genes, metal transporter genes and plant chelating peptide synthase genes. Research has shown that *WRKY74* in wheat is significantly upregulated under copper stress and enhances the plant’s antioxidant capacity through the expression of antioxidant enzyme genes [32]. *WRKY37* in *Oryza sativa* is induced by copper deficiency and positively regulates the expression of copper transport genes, thereby promoting copper uptake and distribution [22]. Although no similar studies have yet been conducted in *Platycodon grandiflorus*, given the evolutionary conservation of the WRKY family and experimental evidence of its differential expression under abiotic stress, it is reasonable to infer that certain *PgWRKY* members in *Platycodon grandiflorus* may also specifically sense copper stress signals and be phosphorylated and activated by the upstream MAPK cascade, thereby initiating the expression of downstream genes related to antioxidant defence systems and metal homeostasis, thus establishing a complete transcriptional regulatory network spanning from stress signal perception to the execution of physiological functions.

### 5.5. Coordination and Cooperation Across All Channels

It is important to recognise that the response pathways described above do not operate in isolation, but rather exhibit synergistic characteristics across temporal and spatial scales as the stress response progresses. In the early stages of stress, the cell wall barrier and cell surface signal detection are the first to be activated [83]; Subsequently, the enzyme-mediated antioxidant system and endogenous antioxidants are rapidly activated, regulating ROS levels; at the same time, the metal chelation system begins to function, reducing the concentration of free copper ions [78]; transcriptional regulatory networks, meanwhile, coordinate gene expression patterns over longer time scales, driving the reprogramming of cellular states. This temporal coordination of multi-level defence mechanisms enables *Platycodon grandiflorus* to maintain intracellular homeostasis as much as possible under copper stress. However, every defence system has its functional limits. When copper stress is too intense or prolonged, the cell wall’s binding capacity may reach saturation; antioxidant enzymes may be inactivated due to protein oxidation; and the synthesis of plant chelating peptides may be impeded by glutathione depletion, ultimately leading to the accumulation of oxidative damage and exacerbated metabolic disorders. The trend observed in *Platycodon grandiflorus* under cadmium and mercury stress—where superoxide dismutase (SOD) activity initially rises before declining, whilst malondialdehyde (MDA) levels continue to rise—is a microcosm of this dynamic process, from the activation to the collapse of the defence system [33]. The increase in electrolyte permeability and malondialdehyde content in *Platycodon grandiflorus* cells under aluminium stress also confirms this pattern [84].

## 6. Future Perspectives

### 6.1. Mechanisms of Structural Remodelling of the Cell Wall Barrier

The cell wall acts as the first line of defence against copper ions, but its role has so far been inferred indirectly based solely on the identification of gene families. Future research should directly measure changes in the content and monomer ratios of pectin and lignin in the cell wall under copper stress, and, in conjunction with the subcellular localisation of copper ions, clarify the binding sites and saturation thresholds of copper within the cell wall. At the same time, functional validation of candidate genes such as *PgCCoAOMT* should be conducted using hairy root or heterologous expression systems to verify whether lignin deposition constitutes an active defence strategy.

### 6.2. Copper Uptake and Transport, and the Glutathione-Plant Chelating Peptide Detoxification Pathway

*Platycodon grandiflorus* roots exhibit a strong capacity to accumulate copper, but the transporters involved in copper uptake (such as COPT, ZIP and YSL) remain entirely unknown. Future work should involve the genome-wide identification of members of these families, and the validation of their transport activity and ion selectivity through yeast heterologous complementation assays. Concurrently, it is necessary to determine the changes in glutathione (GSH) and plant chelating peptides (PCs) under copper stress, to block GSH synthesis using inhibitors to verify the necessity of this pathway in copper detoxification, and to conduct competition experiments involving elements such as iron and zinc to elucidate the effects of ion interactions on the antioxidant system.

### 6.3. Validation of the Regulatory Function of the Transcription Factor PgWRKY

Although 42 PgWRKY genes have been identified in the *Platycodon grandiflorus* genome, their functions have not yet been validated under copper stress. Future work should first involve screening for PgWRKY members that exhibit a significant response to copper, followed by validation using molecular interaction techniques (such as yeast one-hybrid assays and EMSA) to determine whether they bind to the promoters of antioxidant enzymes or copper transporter genes. Finally, the target genes should be overexpressed or silenced in trichome roots or heterologous systems to establish a chain of evidence linking expression to upstream and downstream regulation.

### 6.4. Copper Accumulation and Quality Control

Future studies should screen different germplasms of *Platycodon grandiflorus* to identify low-copper-accumulating genotypes. Quality control standards should move beyond total copper content and consider copper speciation. Post-harvest processing methods—such as washing, soaking, fermentation, or heat treatment—should be tested for their ability to reduce copper levels in the medicinal roots without degrading bioactive compounds (e.g., platycodin D, polysaccharides).

### 6.5. Exogenous Strategies to Minimise Copper Entry into Plants

In addition to investigating the intrinsic stress mechanisms underlying *Platycodon grandiflorus*’s response to copper stress, future research should also consider integrating exogenous intervention strategies, such as reducing the bioavailability of copper in the soil or directly inhibiting or controlling copper uptake by the root system. Specific measures include the application of soil amendments (such as biochar, lime or silicate materials) to immobilise Cu^2+^ through adsorption or precipitation, thereby reducing the concentration of exchangeable copper in the rhizosphere; or the use of copper uptake inhibitors and root-tip-specific transporter blockers, as well as Co-cultivation with mycorrhizal fungi or actinomycetes.

## 7. Conclusions

Copper is an essential trace element for plant growth, yet excessive soil copper triggers copper stress. It harms plants by inducing reactive oxygen species burst, disrupting nutrient homeostasis and inhibiting photosynthesis. As a medicinal and edible root-used herb, *Platycodon grandiflorus* readily accumulates copper via roots, which impairs its growth and medicinal value. This plant establishes a multi-layered defense network against copper toxicity: cell walls act as physical barriers, while endogenous polysaccharides and saponins, together with antioxidant enzymes and the glutathione–phytochelatin system, alleviate oxidative damage. Transcription factors such as PgWRKY also mediate stress signal transduction and gene expression. Current research on its copper stress response remains insufficient, with key molecular mechanisms unclarified. Further studies should explore regulatory pathways, screen low-copper-accumulating germplasms and develop targeted regulation strategies. The findings will support safe cultivation, quality control and breeding of heavy metal-tolerant medicinal plants in copper-contaminated areas.

## Data Availability

No new data were created or analyzed in this study. Data sharing is not applicable to this article.
